# Effects of Italian Smoking Regulation on Rates of Hospital Admission for Acute Coronary Events: A Country-Wide Study

**DOI:** 10.1371/journal.pone.0017419

**Published:** 2011-03-02

**Authors:** Francesco Barone-Adesi, Antonio Gasparrini, Loredana Vizzini, Franco Merletti, Lorenzo Richiardi

**Affiliations:** 1 Cancer Epidemiology Unit, CeRMS and CPO-Piemonte, University of Turin, Turin, Italy; 2 Department of Social and Environmental Health Research, London School of Hygiene and Tropical Medicine, London, United Kingdom; Yale University School of Medicine, United States of America

## Abstract

**Background:**

Several studies have reported a reduction in acute coronary events (ACEs) in the general population after the enforcement of smoking regulations, although there is uncertainty concerning the magnitude of the effect of such interventions. We conducted a country-wide evaluation of the health effects of the introduction of a smoking ban in public places, using data on hospital admissions for ACEs from the Italian population after the implementation of a national smoking regulation in January 2005.

**Methods and Findings:**

Rates of admission for ACEs in the 20 Italian regions from January 2002 to November 2006 were analysed using mixed-effect regression models that allowed for long-term trends and seasonality. Standard methods for interrupted time-series were adopted to assess the immediate and gradual effects of the smoking ban. Effect modification by age was investigated, with the assumption that exposure to passive smoking in public places would be greater among young people. In total, 936,519 hospital admissions for ACEs occurred in the Italian population during the study period. A 4% reduction in hospital admissions for ACEs among persons aged less than 70 years was evident after the introduction of the ban (Rate Ratio [RR], 0.96; 95% Confidence Interval [CI], 0.95–0.98). No effect was found among persons aged at least 70 years (RR 1.00; 95% CI 0.99–1.02). Effect modification by age was further suggested by analyses using narrower age categories.

**Conclusions:**

Smoke-free policies can constitute a simple and inexpensive intervention for the prevention of cardiovascular diseases and thus should be included in prevention programmes.

## Introduction

Second hand smoke causes several diseases, including lung cancer and cardiovascular and respiratory diseases [1]. In recent years, the mounting evidence about the adverse effects of second hand smoke prompted several countries to introduce smoking bans in indoor public places such as public venues and workplaces. Several studies found a reduction in rates of hospital admissions for acute cardiovascular diseases in the general population after the enforcement of smoking bans in indoor public places. However there is still uncertainty on the magnitude of this reduction, with estimates ranging from approximately 0 up to 70% [2], [3], [4], [5], [6]. Sampling variations, different lengths of follow up, changes in active smoking, differences in the prevalence of active and passive smoking in the studied populations and misspecification of the underlying long term-trends, have all been invoked to explain the differences in the effect estimates [3], [4], [7], [8]. A thorough assessment of the health effects of smoking regulations could support the introduction of comprehensive smoking regulations in countries such as China (home to a third of the world's smokers), that do not currently have such policies [9].

On 10 January 2005, Italy became the third European country to ban smoking in all indoor public places, including cafes, bars, restaurants, and discos [10]. This legislation followed earlier restrictions in 1975 for hospital wards, schools, cinemas, and public transports and, in 1995, for public administrations [10]. The smoking ban was enforced on the same day across the 20 regions of the country and achieved very high compliance [11]. Four previous studies conducted in Italy at the city or regional levels have evaluated changes in the rates of hospital admissions for acute coronary events (ACEs) in the first months after the introduction of the legislation [4], [12], [13], [14]. Reported results ranged between no effect and an 11% reduction of the rates of hospital admissions for ACEs among peoples under 65 years of age [4]–[14]. The short period of observation after the introduction of the national regulation precluded researchers to evaluate whether the effect of the ban was stable over time [4], [12], [13], [14]. It is also still not clear whether the introduction of the ban has had the same effect in the whole country. In order to answer these questions, we conducted the first country-wide evaluation of the health effects of the introduction of a smoking ban in public places, using data on hospital admissions for acute coronary events in the Italian population (about 60 million inhabitants) for a period of five years, three years before and two years after the introduction of the ban.

## Methods

### Hospital admissions data

Hospital admissions for acute coronary events (ACEs) occurring in Italy between 2002 and 2006 were obtained from the National Hospital Discharge Database in the form of anonymous individual records that included age, dates of admission and discharge, diagnosis at discharge, gender and region of residence. The National Hospital Discharge Database was established in 1994 and records information on all hospitalisations occurring among Italian residents. The quality of registration has gradually improved over the first five years of the Registry, which is currently regarded as highly complete [15]. The National Hospital Discharge Database is publicly available. We identified hospital admissions for ACEs selecting all records with a primary discharge diagnosis of either acute myocardial infarction (ICD-9-CM code: 410 [16]) or other acute and sub-acute forms of ischemic heart disease (ICD-9-CM code: 411[16]).

Population figures by gender, five-year age groups, calendar year and region were obtained from the National Statistical Office [17].

### Statistical analysis

We analysed rates of admission for ACEs to compare the periods before and after the ban, adjusting for seasonality and long-term trends. Models were based on time-series of monthly hospital admissions in the 20 Italian regions during the years 2002–2006, although December 2006 was not included to avoid a loss of information for patients admitted in December 2006 and discharged in 2007. The series of monthly counts were assumed to follow a Poisson distribution allowing for overdispersion. To account for the fact that long-term trends may have varied among the 20 regions we used mixed-effect regression models in which fixed coefficients described the national trend while random coefficients accounted for region-specific deviations. Preliminary analyses suggested a linear trend at a national level but not for the regional deviations (data not shown). Thus, we chose a parsimonious model with a simple linear function of time for the fixed effects and a cubic function for the random effects.

We modelled the seasonal variations in hospital admissions for ACEs using harmonic functions of time [18]. We adopted standard methods for interrupted time-series to assess the effects of the smoking ban [19]: the immediate effect of the ban was modelled as a step function, including an indicator variable that changed in January 2005, while the gradual effects were studied with an interaction term between the effect of the ban and time.

Several studies have found different effects of the smoking bans among younger and older persons [7], [12], [13], [14], [20]. This could be partially explained by the fact that the population prevalence of exposure to passive smoking in public places varies with age [21], [22]. Therefore, we decided a-priori to analyse people aged below and above 70 years.

We conducted several secondary analyses. First, we used six age classes (less than 40 years; 40 to 49; 50 to 59; 60 to 69; 70 to 79; at least 80 years) to further investigate the possible effect modification introduced by age. Second, we explored possible geographical heterogeneities, evaluating three macro areas: Northern, Central and Southern Italy. We also considered annual all-cause hospitalization rates for Italian residents as an adjustment factor to take into account that changes in the occurrence of acute coronary events may reflect the overall trends of hospital use. Finally, we stratified the effect of the ban by hospital discharge diagnosis (i.e., “acute myocardial infarction” or “other acute and subacute forms of ischemic heart disease”) to investigate the role of possible changes in the diagnostic criteria of acute myocardial infarction during the study period [23].

Analyses were carried out using the software R (Team R Development Core) and Stata (StataCorp, College Station, TX). All tests were two-sides and performed at the 5% level of statistical significance.

## Results

A total of 936,519 hospital admissions for acute coronary events (ACEs) occurred in the

Italian population during the study period. Of these, 371,687 events occurred after the introduction of the smoking ban in January 2005.

**Figure 1:**
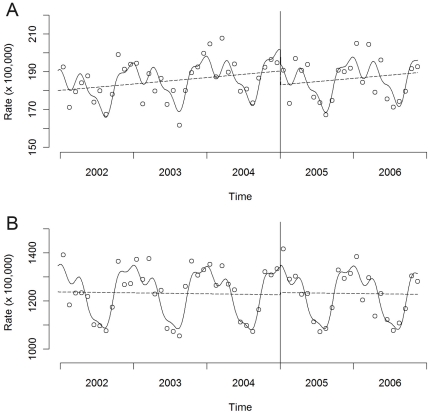
Hospital admissions for Acute Coronary Events (ACEs) in Italy during the period 2002–2006. Observed (circles) and predicted (solid lines) standardised rates among persons under 70 years of age (A) and persons aged at least 70 years (B). The dashed lines represent the deseasonalised trend of ACEs before and after the introduction of the national smoking regulation.

Table 1 displays the number of acute coronary events and the age-standardised admission rates by calendar year. These data are depicted in Figure 1 on a monthly basis together with the predicted regression curves. We observed a decline in rates for acute coronary events among people under 70 years of age immediately after the enforcement of the national smoking ban, while no reduction was observed among older people.

**Table 1:** Number of hospital admissions for acute coronary events in Italy, and age-standardised rates (cases/100,000) by year and age group, 2002–2006.

Age (years)	Gender	2002		2003		2004		2005		2006	
		No. of cases	Rate*	No. of cases	Rate*	No. of cases	Rate*	No. of cases	Rate*	No. of cases #	Rate* †
<70	Men	71320	253	71337	250	74644	257	72742	248	67775	250
	Women	19219	60	19435	61	20563	64	19974	61	18617	62
≥70	Men	50858	1714	53290	1737	53676	1706	54563	1698	50383	1668
	Women	41459	849	44017	872	45014	878	46075	876	41558	843

*Age-standardised according to the European Standard Population [33].

†December 2006 was not included in the calculations to avoid loss of information for patients admitted in December 2006 and discharged in 2007.

**Figure 2:**
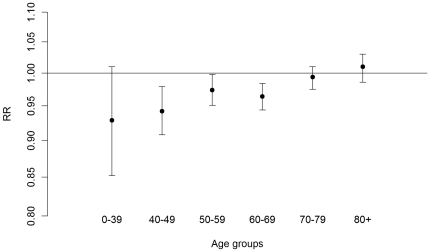
Effect of the smoking ban on different age groups. Rate ratios and 95% confidence intervals of hospital admissions for acute coronary events during the ban period compared with the pre-ban period by age groups. Results adjusted for long-term trends and seasonality. Italy, 2002–2006.

The rate ratio (RR) for all ages for the ban period (January 2005-November 2006) compared with the pre-ban period (January 2002-December 2004) was 0.98 (95% confidence interval [95% CI] 0.97–1.00) (data not shown in Tables). Results for the immediate effects of the ban stratified by age and gender are summarised in Table 2. Among people under 70 years of age, the RR of ACEs for the ban period compared with the pre-ban period was 0.96 (95% CI: 0.95–0.98). The rates of admissions decreased both for men (RR: 0.97; 95% CI: 0.95–0.98) and women (RR: 0.95; 95% CI: 0.93–0.98). People over 70 years of age experienced no decrease in admissions for ACEs after the introduction of the ban. A monotonic effect modification by age on the effect of the ban is further suggested by analyses based on narrower age categories (Figure 2).

**Table 2:** Rates of admission for acute coronary events during the ban period compared with the pre-ban period by gender and age.

	<70 years	> = 70 years
Gender	RR (95% CI)	RR (95% CI)
Men	0.97 (0.95–0.98)	1.00 (0.98–1.02)
Women	0.95 (0.93–0.98)	1.01 (0.98–1.03)
Overall	0.96 (0.95–0.98)	1.00 (0.99–1.02)

Results adjusted for long-term trends and seasonality. Italy 2002–2006.

RR: Rate Ratio.

95% CI: 95% Confidence Interval.

We did not find evidence of a gradual effect of the ban, as there was no change in the underlying trend in admissions for ACEs after January 2005 among people aged under 70 (p value = 0.60) (Figure 1A). Overall, from January 2005 to November 2006, the model predicts 7,100 fewer admissions for acute coronary events in Italy among people under 70.

In secondary analyses run among people younger than 70 years of age, the effect of the ban was similar in the different geographical areas, (Northern Italy RR: 0.96; 95% CI: 0.94–0.99; Central Italy RR: 0.95; 95% CI: 0.92–0.99; Southern Italy RR: 0.97; 95% CI: 0.94–1.00). Moreover, stratification by type of diagnosis (“acute myocardial infarction” or “other acute and sub-acute forms of ischemic heart disease”) did not alter our results (Table 3). Also adjustment for annual all-cause hospitalization rates did not change the results substantially from the main analysis (RR for both sexes under 70 years of age: 0.96; 95% CI: 0.94–0.98).

**Table 3:** Rates of admission for acute myocardial infarction and other acute and sub-acute forms of ischemic heart diseases during the ban period compared with the pre-ban period, by gender and age.

	Acute myocardial infarction (ICD9 410)		Other acute and sub-acute forms of ischemic heart diseases (ICD9 411)	
	<70	≥70	<70	≥70
Gender	RR (95% CI)	RR (95% CI)	RR (95% CI)	RR (95% CI)
Men	0.97 (0.95–0.99)	1.00 (0.98–1.03)	0.96 (0.93–0.99)	0.98 (0.95–1.01)
Women	0.98 (0.94–1.02)	1.02 (0.99–1.04)	0.92 (0.88–0.98)	0.97 (0.94–1.01)
Overall	0.97 (0.95–0.99)	1.01 (0.99–1.04)	0.95 (0.93–0.98)	0.98 (0.96–1.00)

Results adjusted for long-term trends and seasonality. Italy 2002–2006.

RR: Rate Ratio.

95% CI: 95% Confidence Interval.

## Discussion

In an analysis based on nearly one million hospital admissions, we found a decrease in rates of hospitalisation for acute coronary events (ACEs) among both men and women aged under 70 in the two years following the introduction of a comprehensive smoking ban in Italy. The observed reduction was stable over the study period, was similar in the different geographical areas, and was stronger among younger people.

Several pieces of evidence suggest that the Italian ban has been able to reduce the population exposure to passive smoking. The smoking legislation is almost universally respected, with less than 100 (1.5%) violations observed in about 6,000 checks by the police during the first year after the implementation of the law [24]. Almost 90% of people interviewed in a large national survey in 2005 reported that the smoking ban was observed in bars and restaurants, and 70% reported the same regarding workplaces [11]. Moreover, studies that measured concentrations of environmental nicotine and particulate matter with diameter <2.5 µm in hospitality premises of different Italian cities before and after the smoking ban showed reductions ranging from 70% to 97% [11].

Potential confounding in time series studies is limited to factors that are related to the outcome of interest and change at the time of the intervention [19]. In contrast, temporal changes in the prevalence of individual risk factors (such as hypertension and cholesterol level) shape the underlying long-term trends, and are thus inherently taken into account in time-series studies. To our knowledge, with the exclusion of the national smoking regulation, no nation-wide intervention potentially able to cause a sharp decrease in rates of hospital admissions for ACEs took place in Italy during the study period. By adjusting for seasonality, we took into account most of the potential confounding effect of risk factors that change periodically during the year, including temperature and air pollution. In a previous study conducted among residents in the city of Rome, the estimates of the cardiovascular effects of the Italian smoking regulation were adjusted for a number of possible confounders including apparent temperature, flu epidemics and the air concentration of PM _10_ [14]. Interestingly, reported crude and adjusted estimates were almost identical [14]. Moreover, most of these risk factors mainly affect elderly adults [25], while the reduction in risk observed in the present study mainly occurred among younger people (Figure 2).

Although a fraction of patients with acute coronary events dies before reaching a hospital, it is likely that mortality outside of the hospital has not changed substantially from 2002 to 2006. Again, the Rome study supports this assumption, as Cesaroni and colleagues found similar effects of the smoking regulation on mortality rates and hospital admissions [14]. Some researchers have suggested that changes in the diagnostic criteria for acute myocardial infarction introduced by the European Society of Cardiology and the American College of Cardiology in 2000 may have induced an apparent increase in the rates of hospital admission thereafter [23]. However, some studies have shown that the impact of these new diagnostic criteria was lower in Italy than in other countries, especially when acute myocardial infarction and other acute and sub-acute forms of ischemic heart disease are grouped together, as was the case in our study [26].

Growing evidence indicates that both active and passive smoking increases cardiac risk through both chronic (atherosclerosis) and acute (platelet activation and endothelial dysfunction) pathways [27]. A reduction in the admissions for ACEs shortly after the introduction of the smoking ban is consistent with findings from laboratory and epidemiological studies, which report that the acute effects of both active and passive smoking disappear within a short time after cessation of the exposure [27], [28].

Smoking regulations decrease exposure to passive smoking and may indirectly decrease active smoking. However, simulation studies [12] and empirical evidence on the effect of the introduction of bans among smokers and non-smokers [7], [20], [29] suggest that most of the observed effects of smoking regulations can be attributed to decrease in passive smoking. In a recent simulation study, we estimated that a 4% reduction in hospital admissions for ACEs could be obtained with a 15% to 60% decrease in the exposure to passive smoking from any source among the general population [8]. Indeed, decreases of this magnitude have been found in population surveys that measured changes in the levels of salivary cotinine among non-smokers, following the introduction of smoking regulations [8]. Moreover, the prevalence of exposure to passive smoking in public places is reported to be higher among the youngest age groups, which is consistent with our finding of a decreasing effect of the smoking ban with age [21], [22].

We found a lower decrease in admissions for ACEs than some previous studies evaluating the effect of the introduction of smoking regulations in other countries [3], [7]. However, most of these studies did not fully account for underlying trends in hospital admissions; thus, they may have overestimated the real effect of smoking bans [4], [30]. Two large studies conducted in England and the New York State adjusted for long-term trends and found decreases in admissions for acute myocardial infarction of 2% and 8% respectively, consistent with our estimates [30], [31]. It should also be noted that comprehensive smoking bans were introduced in England, Italy and New York State when many public places and work environments were already smoke-free [30], [31]. For example, in Italy, smoking in public administration buildings was already banned in 1995 (even if the level of compliance with this law was not high) [10]. Therefore, the low levels of exposure to passive smoking in some work environments before the introduction of comprehensive smoking regulations may have limited their potential benefits.

Assuming that the introduction of a smoking regulation changes the rates of hospital admissions for ACEs and mortality rates for coronary heart disease in the general population to a similar extent [14], the 4% decrease in events that we found can be compared with the effects of other interventions to decrease mortality by coronary heart disease and with the effects of population-wide changes in the prevalence of cardiovascular risk factors. In a recent study, Palmieri and colleagues used the IMPACT model to explain the decrease in coronary heart disease mortality in Italy between 1980 and 2000 [32]. Changes in prevalence of active smoking (from 31.7% in 1980 to 21.8% in 2000) were responsible for a 3.7% reduction in mortality for coronary heart disease during the study period. Similar effects were estimated for the introduction of novel therapies, such as the early medical treatment of acute myocardial infarction (−4.9%), hypertension treatments (−1.5%) and the use of statins for primary prevention (−2.7%) [32]. Thus the magnitude of the effect of a smoking regulation is comparable with those of other interventions, but such effects are obtained immediately and with limited cost to the community [11].

### Conclusions

The results of this study, which was carried out on a large population and over a long period of observation, suggest that smoke-free policies may result in a short-term reduction in hospital admissions for ACEs. This finding has important implications for public health. Smoking regulations represent a simple, effective and inexpensive intervention for the prevention of cardiovascular diseases and should be taken into account in prevention programmes.
